# Using CorvisST tonometry to assess glaucoma progression

**DOI:** 10.1371/journal.pone.0176380

**Published:** 2017-05-04

**Authors:** Masato Matsuura, Kazunori Hirasawa, Hiroshi Murata, Shunsuke Nakakura, Yoshiaki Kiuchi, Ryo Asaoka

**Affiliations:** 1 Department of Ophthalmology, University of Tokyo Graduate School of Medicine, Tokyo, Japan; 2 Orthoptics and Visual Science, Department of Rehabilitation, School of Allied Health Sciences, Kitasato University, Kanagawa, Japan; 3 Department of Ophthalmology, Saneikai Tsukazaki Hospital, Himeji, Japan; 4 Department of Ophthalmology and Visual Science, Hiroshima University, Hiroshima, Japan; University of Tennessee Health Science Center, UNITED STATES

## Abstract

**Purpose:**

To investigate the utility of the Corneal Visualization Scheimpflug Technology instrument (CST) to assess the progression of visual field (VF) damage in primary open angle glaucoma patients.

**Method:**

A total of 75 eyes from 111 patients with primary open-angle glaucoma were investigated. All patients underwent at least nine VF measurements with the Humphrey Field Analyzer, CST measurements, axial length (AL), central corneal thickness (CCT) and intraocular pressure (IOP) with Goldmann applanation tonometry (GAT). Mean total deviation (mTD) progression rates of the eight VFs, excluding the first VF, were calculated and the association between progression rate and the other listed measurements was analyzed using linear regression, and the optimal to describe mTD progression rate was selected based on the second order bias corrected Akaike Information Criterion (AICc) index.

**Results:**

VF progression was described best in a model that included CST parameters as well as other ocular measurements. The optimal linear model to describe mTD progression rate was given by the equation: -8.9–0.068 x mean GAT + 0.68 x A1 time + 0.31 x A2 time -0.39 x A2 length– 1.26 x highest deformation amplitude.

**Conclusion:**

CST measurements are useful when assessing VF progression in glaucoma patients. In particular, careful consideration should be given to patients where: (i) an eye is observed to be applanated fast in the first and second applanations, (ii) the applanated area is wide in the second applanation and (iii) the indentation is deep at the maximum deformation, since these eyes appear to be at greater risk of VF progression.

## Introduction

Glaucoma is the leading cause of irreversible blindness worldwide with approximately 60 million people suffering from the disease[1]. In glaucoma, intraocular pressure (IOP) should be adequately controlled to avoid visual field (VF) deterioration.[2–10] Goldmann applanation tonometry (GAT) is widely considered the gold standard method to measure IOP in glaucoma patients. However, a limitation of GAT is that its measurements are influenced by corneal properties, including central corneal thickness (CCT).[11–23] Furthermore, several studies have suggested that the progression of glaucoma is related to the magnitude of CCT itself,[4,24] although more recent studies[25] have revealed that other corneal measurements, namely corneal hysteresis (CH) and corneal resistance factor (CRF), measured with the Ocular Response Analyzer (ORA, Reichert Ophthalmic Instruments, Depew, NY, USA), are more closely related to the progression of glaucoma. Thus, it is clear that the biomechanical properties of the cornea are a risk factor for the progression of glaucomatous neuropathy. Corneal biomechanics can now be captured using the Corneal Visualization Scheimpflug Technology instrument (Corvis ST tonometry: CST; Oculus, Wetzlar, Germany). In CST, the corneal movement during the application of a rapid air-puff is captured using an ultra-high-speed Scheimpflug camera. The biomechanical properties of the cornea can then be assessed both visually and quantitatively.[26] However, to date, the relationship between CST parameters and glaucomatous VF progression has not been investigated in detail. Therefore, the purpose of the current study is to investigate the importance of CST parameters on VF deterioration in patients with primary open angle glaucoma (POAG).

## Method

The study was approved by Research Ethics Committee of the Graduated School of Medicine and Faculty of Medicine at The University of Tokyo and Hiroshima University Hospital (Hiroshima, Japan). Written consent was given by patients for their information to be stored in the hospital database and used for research. This study was performed according to the tenets of the Declaration of Helsinki.

## Subjects

A total of 111 eyes from 75 POAG patients (39 males and 36 females) were included in this study. All patients had at least nine VF tests with the Humphrey Field Analyzer II (HFA, Carl Zeiss Meditec Inc, Dublin, CA), with 24–2 or 30–2 and SITA standard programme. Reliable VFs were defined as Fixation loss (FL) rate <20% and also False positive (FP) rate <15% following the criteria used in the HFA software. The first VF measurement was discarded to mitigate the learning effect. Consequently, eight VFs were used to measure the rate of progression; this number was specifically chosen as we have recently reported that a precise assessment of VF progression can be achieved using VFs with this volume.[27–31]

Patients with abnormal eye-related findings (except for OAG) on biomicroscopy, gonioscopy and funduscopy were excluded. In addition, eyes that experienced any ocular surgery, including trabeculectomy and cataract surgery were also carefully excluded. Only subjects aged 20 years or older were included and eyes with IOP>25 mmHg or contact lens wearers were excluded. Both eyes of a patient were included in the study, if they were both eligible.

### Corvis ST tonometer measurements

CST was performed within 180 days after the final VF measurement. CST (software version; 1.2r1092) measurements were repeated three times and all measurements had sufficient reliability, as indicated by the “OK” quality index displayed on the monitor. Patients were given at least one minute interval to rest between each measurement.

As described in detail elsewhere,[26] CST captures a sequence of images (4330 images per second) of corneal deformation, and various parameters of deformation amplitude, applanation length and corneal velocity are quantified.‘A1/A2 time’ measurements capture the time from the initiation of the air puff to the first (inward corneal movement) or second applanation (outward corneal movement); ‘A1/2 length’ is the length of the applanated flat cornea surface at the first / second applanation; ‘A1/2 velocity’ is the velocity of corneal apex movement during the first / second applanation; ‘A1/2 deformation amplitude’ is the magnitude of the movement of the corneal apex at the first / second applanation; ‘peak distance’ is the distance between the two surrounding peaks on the cornea at the highest concavity; ‘highest deformation amplitude' is the magnitude of the movement of the corneal apex at the highest concavity: ‘highest concavity time’ is the duration from the initiation of the air puff to the highest concavity of the deformation of cornea: ‘radius’ is the central curvature radius at the highest concavity.

### Other measurements

CCT was measured using CST three times and the average was used in subsequent analyses. The mean and standard deviation (SD) of GAT during the follow up period were calculated. Axial length (AL) was measured using the IOL Master (Carl Zeiss Meditec).

### VF data

The mean total deviation (mTD) was calculated from the 52 test points in the 24–2 HFA VF. The progression rate of mTD, across the eight VFs, was then calculated using linear regression against time.

### Statistical analysis

The relationship between ocular/systemic parameters (age, mean GAT, SD of GAT, CCT, AL, and mTD in the initial VF), CST parameters (A 1/2 time, A 1/2 length, A 1/2 velocity, A 1/2 deformation amplitude, highest deformation amplitude, highest concavity time, peak distance, and radius) and mTD progression rate was investigated using linear mixed modelling. In the linear mixed model, a patient is included as a random effect so that both eyes of a patient are appropriately included.

First a linear mixed model was built using only the six ocular/systemic parameters (‘Model_A_’) and the optimal to describe mTD progression rate was selected based on the second order bias corrected Akaike Information Criterion (AICc) index. Next, a second model was built using ocular/systemic parameters as well as CST measurements (‘Model_B_’). In a linear regression model, the degrees of freedom decreases as the number of variables increases, hence model selection methods should be used when the number of variables is large.[32,33] The AICc (the corrected form of the AIC) was used since this gives an accurate estimation even when the sample size is small.[34]

Any reduction in AICc suggests an improved model, but the probability that one particular model is the model that minimizes ‘information loss’ is calculated as follows. When there are *n* candidate models and the AICc values of those models are AICc_1_, AICc_2_, AICc_3_, …, AICc_*n*_. Let AICc_min_ be the minimum of those values, exp((AICc_min_ − AICc_*i*_)/2) is the relative probability that the *i*th model minimizes information loss.[35] All statistical analysis were performed using the statistical programming language ‘R’ (R version 3.2.3;The foundation for Statistical Computing, Vienna, Austria)

## Results

Characteristics of the study subjects are summarized in Table 1. The mean ± standard deviation (SD) [range] age was 63.3±9.7 [43 to 85] and 39 patients were male and 36 patients were female. Eight VFs were measured over an average of 2364.0±872.8 [630 to 6881] days. GAT was measured 27.9±8.4 [8 to 69] times during the follow up period (between initial VF and eighth VF). The mean GAT during the follow up period was 13.4±2.2 [8.9 to 20.2] mmHg with an SD value of 1.6±0.5 [0.8 to 3.6]. The mTD progression rate was -0.28±0.4[2.7 to 1.4] dB/year. A summary of CST measurements are shown in Table 2. Among 111 eyes of 75 patients, 65 eyes of 44 patients used prostaglandin analogues throughout the observation period and 31 eyes of 22 patients used prostaglandin analogues at least once in the observation period.

**Table 1:** Subject demographics.

Variables	Value
age, (mean ±SD) [range], years old	63.3 ± 9.7 [43 to 85]
Male / Female	39 / 36
Right / Left	56 / 55
Mean GAT, (mean ±SD) [range], mmHg	13.4 ± 2.2 [8.9 to 20.2]
AL, (mean ±SD) [range], mm	25.1 ± 1.6 [22.3 to 29.2]
CCT, (mean ±SD) [range],μm	531.8 ± 36.6 [458.3 to 645]
mTD, (mean ±SD) [range], dB	-6.9 ± 6.5 [-27.0 to 3.9]

sd: standard deviation, GAT: intraocular pressure measured with Goldmann tonometry, AL: axial length, CCT: central corneal thickness, mTD: mean of total deviation values.

**Table 2:** Measured CST parameters.

CST parameter	Value (mean±sd) [range]
A1 time (ms)	7.2 ±0.29 [6.5 to 8.4]
A1 length (mm)	1.7±0.079 [1.4 to 1.8]
A1 velocity (m/s)	0.16±0.015 [0.099 to 0.20]
A1 deformation amplitude (mm)	0.12±0.0082 [0.11 to 0.16]
A2 time (ms)	21.9±0.47 [20.9 to 23.2]
A2 length (mm)	1.7±0.23 [0.83 to 2.2]
A2 velocity (m/s)	-0.39±0.079 [-0.16 to -0.63]
A2 deformation amplitude (mm)	0.41±0.073 [0.57 to 0.25]
highest deformation amplitude (mm)	1.1±0.11 [0.82 to 1.3]
highest concavity time (ms)	16.9±0.58 [15.4 to 18.4]
Peak distance (mm)	3.5±0.95 [2.1 to 5.5]
Radius (mm)	7.5±0.85 [5.9 to 10.3]

sd: standard deviation, CST: Corvis ST tonometry.

The equation of Model_A_ was: mTD progression rate = 0.25–0.0085 * age (AICc = 155.6); thus mean GAT, SD of GAT, CCT, AL and mTD in the initial VF were not selected as predictors. The AICc values with each CST parameter are shown in Table 3. A decrease in AICc was observed with all CST parameters compared to Model_A_.

**Table 3:** Correlation between CST parameters and visual field progression rate.

	Coefficient	standard error	AICc
A1 time (ms)	0.24	0.14	149.7
A1 length (mm)	0.053	0.54	149.7
A1 velocity (m/s)	-4.3	2.8	144.1
A1 deformation amplitude (mm)	6.4	5.2	143.7
A2 time (ms)	-0.017	0.091	153.3
A2 length (mm)	-0.22	0.18	150.5
A2 velocity (m/s)	0.59	0.54	148.6
A2 deformation amplitude (mm)	-0.26	0.58	149.4
highest deformation amplitude (mm)	-0.63	0.39	147.8
highest concavity time (ms)	0.013	0.0740	153.7
Peak distance (mm)	-0.060	0.045	153.0
Radius (mm)	0.070	0.050	152.6

CST: Corvis ST tonometry.

The equation for Model_B_ was: mTD progression rate = -8.9–0.068 x mean GAT + 0.68 x A1 time + 0.31 x A2 time -0.39 x A2 length– 1.26 x highest deformation amplitude (AICc = 137.3). The probability that Model_B_ is optimal (minimizes information loss) compared to Model_A_ was 99.99%.

## Discussion

In the current study CST measurements were carried out in 111 eyes of 75 patients with POAG. VF progression was measured over a period spanning approximately 7 years. Notably, VF progression could be modelled more accurately by including CST parameters in a linear model. This optimal model also included mean GAT during the follow up (higher IOP indicates faster progression), A1 time (shorter time indicates faster progression), A2 time (shorter times suggests faster progression), A2 length (longer length implies faster progression), highest deformation amplitude (higher amplitude signifies faster progression).

The first model, Model_A_, (the optimal model without CST parameters) did not include mean GAT, despite many previous studies, including randomized controlled trials (RCTs), [3,36–39] that have suggested IOP is a very important factor for managing the progression of glaucoma. The lack of mean GAT in the model is probably because the current study analyzed data obtained from a real world clinic where the management of IOP is decided by clinicians according to the progression of glaucoma. As a result, the direct, at least, effect of IOP on glaucoma progression may be masked, which was also the case in our very recent multi-central study.[40] In a recent paper we found that SD of IOP was related to the progression of glaucoma,[40] but in the current study only age was selected among the basic ocular/systemic factors. Age is an important risk factor for the progression of glaucoma[4,41–43] even in our recent study based on a real world clinical dataset.[40] Interestingly, however, age was no longer included in Model_B_ (the optimal model that included CST parameters), but a number of CST parameters were included: A1 time, A2 time, A2 length and highest deformation amplitude. As shown in our previous report,[44] age is correlated with shorter A1 time, shorter A2 time, and also deeper highest deformation amplitude. The values of the coefficients of these parameters observed in Model_B_ follow the pattern of aging, which may suggest that glaucomatous VF progression is more accurately described by changes in corneal biomechanics using CST parameters associated with age rather than using age directly. This is clinically very important because an eye exhibiting these properties has a higher risk of progression, regardless of the patient’s age.

Many previous studies have demonstrated that thin CCT is a risk factor for the progression of glaucoma[4,11,13,24,45,46], however, CCT was not included in Model_A_, nor was it included in Model_B_. It has recently been suggested that the viscoelastic property of the cornea (corneal hysteresis) is a stronger risk factor for the progression of glaucoma than CCT.[47] In the current study, CST parameters which also measure biomechanical properties of the cornea were selected in Model_B_, and this model was significantly superior to Model_A_ to describe VF progression (the probability that model_A_ is superior to Model_B_ was just 6%). CST parameters capture detailed biomechanical properties and thus may better describe the progression of glaucoma than other simpler properties such as CCT.

The stage of VF damage may[4,41,48] or may not[42,49,50] be related to faster VF progression. In the current study, mTD in the initial VF was not included in either model, however, the study population consisted of patients with a relatively early stage of glaucoma (mean mTD = -6.2 dB) so different results could be observed in eyes with more advanced glaucoma. A future study should be carried out to investigate the effect of initial VF damage, and the relationship with CST parameters, in eyes with advanced stage glaucoma.

**Fig 1:**
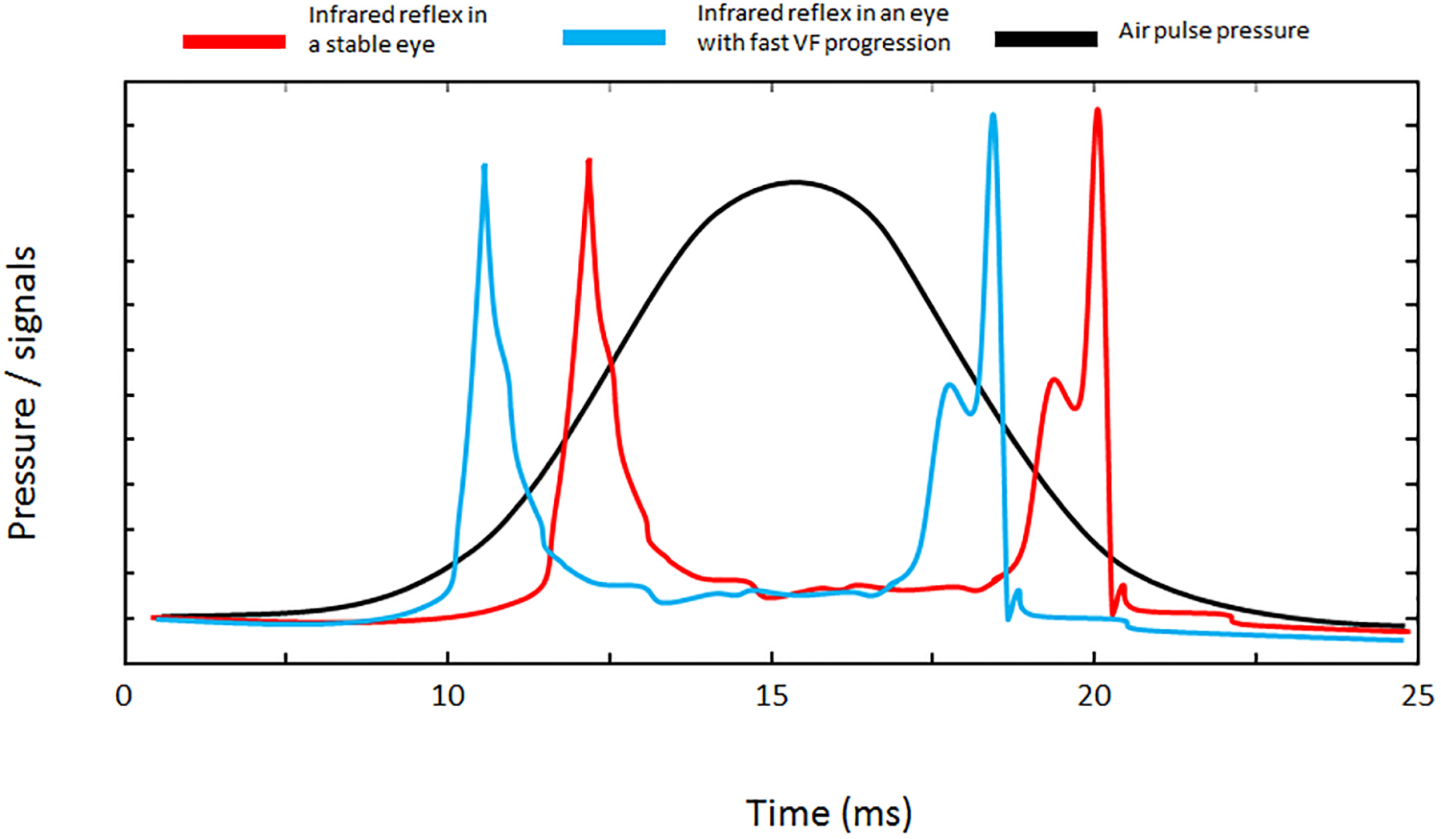
Air–pulse pressure and infrared signal reflected from the corneal surface at A1 and A2 times. The infrared signal reflected from the corneal surface in an eye with fast A1 and A2 time shifts to left side (please see the comparison of the red and blue lines).

As suggested by Model_B_ eyes that are quickly applanated at the first and second applanations (short A1 and A2 time) were more likely to show fast progression. Fig 1 shows the air–pulse pressure and infrared signal reflected from the corneal surface at A1 and A2 times. The infrared signal reflected from the corneal surface in an eye with short A1 and A2 time (an eye more likely to exhibit fast VF progression) shifts to the left; see red and blue lines in Fig 1. In such an eye, the corneal top would start moving backwards while the applied air pulse energy is less accumulated, which would result in it returning to its initial shape more quickly. This suggests that a cornea that is easily deformed is more likely to progress, and thus the biomechanical properties of such an eye may be different to those of a stable eye. The hysteresis of a viscoelastic material is defined as the amount of energy absorption in the ‘loading/unloading’ stress/strain cycle and the magnitude of the energy absorption can be calculated as the area surrounded by the loading and unloading curves.[51] In glaucoma, it has been reported that corneal hysteresis reflects reduced compliance of the lamina cribrosa and thus it may provide further information about glaucoma risk.[52,53] The reason why eyes with low corneal hysteresis are at greater risk for the advancement of glaucoma is not entirely clear, but it may be because these eyes are exposed to greater changes of magnitude in IOP in their daily life (such as postural change,[54] eye lid blinking,[55] ocular pulsatility due to ocular hemodynamics,[56] Valsalva maneuver[57]). It is also possible that an eye with high hysteresis is more likely to absorb these external strains, which would be advantageous to prevent retinal nerve fiber damage at the optic nerve and also retinal ganglion cell loss. A similar hypothesis could be argued regarding strain at the optic disc due to eye movements.[58]

The mechanism to measure A1 time in CST is very similar to that in air-puff tonometers; the time to applanation is measured following an air-puff injection where force increases with time,[59] although the movement of cornea is captured using the reflection of an installed infrared light in air-puff tonometry whereas CST detects the movement of the cornea using the captured images. Thus, A1 time takes on a lower value in an eye with a lower IOP. IOP is an established risk factor of the progression of glaucoma and indeed this study showed that high mean IOP-GAT is related to fast visual field progression. Importantly ‘true’ IOP cannot be measured with any current tonometers; further, it has been reported that IOP readings from air-puff tonometry are greatly decreased in eyes with thin CCT.[21] Thin CCT has been reported to be a risk factor for the progression of glaucoma[4,24] so it would be of interest to investigate whether it remains a risk factor in eyes whose CST-measured IOP readings are lower than in other tonometers less influenced by CCT, such as dynamic contour tonometry.[45]

Eyes experiencing a deep indentation of the cornea with the CST air-puff and eyes with a longer A2 length were at greater risk of fast progression. As CST applies the air pressure with an uniform magnitude to the corneal surface in all measurements, a large highest concavity deformation amplitude value suggests an eye that is easily deformed (fragile). Further, a wide applanated diameter at the second applanation (A2 length) would therefore be associated with a deep highest concavity deformation amplitude. Previous studies have investigated the relationship between some CST parameters and glaucoma. Jung et al. investigated the relationship between highest deformation amplitude and β-zone parapapillary atrophy (βPPA) and reported that a large highest deformation amplitude was associated with a large PPA area.[60] Other studies have compared CST parameters between normal and glaucomatous eyes in a cross-sectional manner, suggesting highest concavity deformation amplitude is lower in glaucoma patients than in normal subjects.[61,62] To the best of our knowledge, the present study is the first to investigate the progression rate of glaucoma and CST parameters using a longitudinal dataset. Furthermore, in the previous studies,[60,61,63] the relationship between corneal deformation amplitude and glaucoma was investigated using a univariate analysis. Higher IOP is associated with the progression of glaucoma, but high IOP is also associated with decreased corneal deformation amplitude due to the resistance effect induced by increased pressure. Indeed, in the current population, low corneal deformation amplitude was significantly related to high IOP (p < 0.001, linear mixed model, data not shown in Results). This strongly implies that the relationship between low corneal deformation amplitude and glaucoma in these previous studies may by biased by the indirect effect of high IOP on low corneal deformation amplitude. In the current study, the influence of corneal deformation amplitude was investigated adjusting for IOP level (multivariable linear mixed model: Model_B_); our results suggest that higher corneal deformation amplitude is related to the progression of glaucoma. However, in the current study, higher corneal deformation amplitude was related to progression in the univariate analysis also (Table 3). The reason for this is not entirely clear, but it could be because our study was carried out in carefully treated glaucoma patients in a real world clinic, and hence the effect of IOP on progression of glaucoma was almost negligible, as shown in our recent report.[40] A limitation of the current study is the lack of a ‘control arm’ of healthy subjects. The effect of CST parameters, considering the importance of IOP in a multivariate analysis, should be investigated in a longitudinal dataset from normal subjects in a future study. A further limitation of the current study is that the effect of anti-glaucomatous eye drops, which are known to change the biomechanical properties of the cornea, could not be controlled for.[63–66] As the patients in the current study were recruited from a real world glaucoma clinic, most patients would be taking eye drops to control their IOP. Thus, a future study should be designed to exclude the effect of the use of anti-glaucomatous eye drops. The studied patients in the current study were treated not basing on the CST parameter values. Different or even reversed findings could be observed when clinicians treat patients considering their CST information.

In conclusion, it appears useful to carry out CST when assessing the progression of glaucomatous VF damage. Careful management is needed in eyes with short A1 time, short A2 time, long A2 length and a large highest deformation amplitude.

## Supporting information

S1 File Data analyzed. (CSV)
